# The Toxic Effects and Mechanisms of CuO and ZnO Nanoparticles

**DOI:** 10.3390/ma5122850

**Published:** 2012-12-13

**Authors:** Ya-Nan Chang, Mingyi Zhang, Lin Xia, Jun Zhang, Gengmei Xing

**Affiliations:** 1 Chinese Academy of Science Key Laboratory for Biomedical Effects of Nanomaterials and Nanosafety, Institute of High Energy Physics, Chinese Academy of Science, Beijing 100049, China; E-Mails: changyn@ihep.ac.cn (Y.-N.C.); zhangmy@ihep.ac.cn (M.Z.); 2 School of Life Science, Beijing Institute of Technology, Beijing 100081, China; E-Mail: xialin@ihep.ac.cn (L.X.)

**Keywords:** CuO, ZnO, nanoparticles, toxic effect, size, solubility, exposure routes, oxidative stress, coordination effects, non-homeostasis effects

## Abstract

Recent nanotechnological advances suggest that metal oxide nanoparticles (NPs) have been expected to be used in various fields, ranging from catalysis and opto-electronic materials to sensors, environmental remediation, and biomedicine. However, the growing use of NPs has led to their release into environment and the toxicity of metal oxide NPs on organisms has become a concern to both the public and scientists. Unfortunately, there are still widespread controversies and ambiguities with respect to the toxic effects and mechanisms of metal oxide NPs. Comprehensive understanding of their toxic effect is necessary to safely expand their use. In this review, we use CuO and ZnO NPs as examples to discuss how key factors such as size, surface characteristics, dissolution, and exposure routes mediate toxic effects, and we describe corresponding mechanisms, including oxidative stress, coordination effects and non-homeostasis effects.

## 1. Introduction

Nanomaterials have gained increasing attention because of their novel properties, including a large specific surface area and high reaction activity [1,2]. Due to rapid development of nanotechnology, nanomaterials with various shapes and diameters have been prepared and used in some industrial products and commodities [3,4,5]. For example, nano-TiO_2_ was used as a cosmetic functional additives to combat aging based on its ability to absorb ultraviolet (UV) light [6], and zinc (Zn) nanomaterials have been used as a highly reactive catalyst in automobile tail gas treatment [7]. Nanomaterials have also been used for various fundamental and practical applications, such as drug delivery, cell imaging, and cancer therapy [8,9,10,11,12].

Metal oxide nanoparticles (NPs) belong to a family of nanomaterials that have been manufactured on a large scale for both industrial and household applications, and they hold promise for future applications. Bibliometric indicators suggest that research into environmental concern of NPs has increased since the first paper on nanoecotoxicology was published in 2006 [13]. Recently, copper oxide (CuO) and zinc oxide (ZnO) NPs (as the representative of industrial and household use, respectively) have been shown to have negative effects on the survival and growth of organisms [14]; these studies suggest that the release of NPs might have negative impacts on both organisms and the environment.

### 1.1. Characteristics of CuO NPs

CuO is the simplest member in the family of Cu compounds and exhibits a range of potential physical properties, such as high temperature superconductivity, electron correlation effects, and spin dynamics [15,16]. As a semiconducting compound with a monoclinic structure, CuO has attracted particular attentions in the field. It possesses useful photovoltaic and photoconductive properties because CuO crystal structures have a narrow band gap [17]. Besides the property, CuO NPs hold novel characteristics. CuO NPs can also improve fluid viscosity and enhance thermal conductivity, and these novel properties make them a potentially useful energy-saving material that can improve the effect of energy conversion [18]. CuO NPs have been applied in different areas, including gas sensors [19], catalysis [20], batteries [21], high temperature superconductors [22], solar energy conversion [23], and field emission emitters [22]. For industrial catalysis, CuO NPs may be able to replace noble metal catalysts for carbon monoxide oxidation [24], which would reduce production cost and improve the catalytic efficiency. The suspension has excellent thermal conductivity and can be used as a heat transfer fluid in machine tools [25]. CuO is much cheaper than silver oxide and can be mixed with polymers more easily to obtain composites with unique chemical and physical properties. Because they can reduce friction [26], and mend worn surfaces, CuO NPs are used as an additive in lubricants, polymers/plastics, and metallic coatings [27]. Moreover, the extremely high surface areas and unusual crystal morphologies endow CuO NPs with antimicrobial activity, and they dose-dependently inhibit *Escherichia coli* strains, but not *Salmonella typhimurium* [28]. This finding paves the way to develop a novel and specific antimicrobial agent [29,30].

### 1.2. Characteristics of ZnO NPs

Zn is essential to life, but it is toxic at high levels. ZnO NPs are widely used as nanosensors [31], UV-absorbers [32], and catalysts [33]. Some studies have reported that ZnO and its NPs have strong absorption abilities for a series of organic compounds and heavy metals [34,35]. Because ZnO NPs are considered safe for humans and they reflect UV light better than micro-particles, they have been widely used as ingredients in cosmetics and modern sunscreens. Information about their safety/toxic effect on skin has continued to increase, but there is a lack of toxicological data [36].

The rising commercial use and large-scale production of engineered NPs may result in unintended exposure to human beings and the environment. In addition to increasing our understanding of NPs’ toxicity, it is necessary to adequately study the properties of CuO and ZnO NPs; there is an urgent need to understand their toxicity to organisms and the environment through the processes of absorption, biodistribution, metabolism, and excretion of nanomaterials *in vivo*. This is important to ensure that their applications are safe and provide helpful information to develop nanomaterial safety standards.

**Figure 1:**
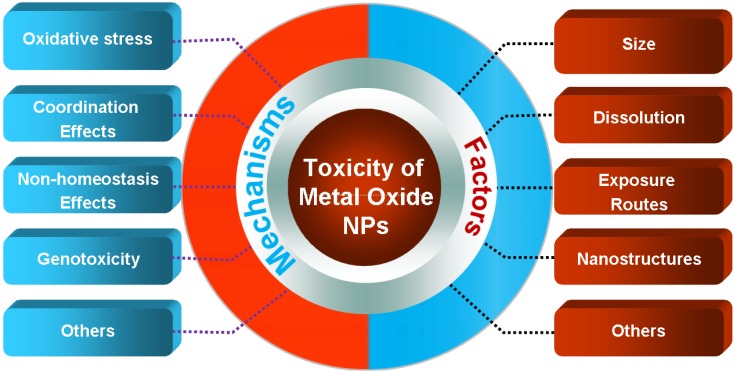
Schematic overview summarizing the toxic effect of CuO and ZnO NPs. The key factors that result in toxicity include particle size, dissolution, exposure routes, and structures, *etc*. The damaging mechanisms include oxidative stress, coordination effects, non-homeostasis, and genotoxicity, *etc*.

This review will provide a brief overview of the toxicity of CuO and ZnO NPs and discuss key factors and toxicity mechanisms (Figure 1).

## 2. Toxicity of CuO and ZnO NPs

### 2.1. Toxicity of CuO NPs

In organisms, Cu is one of indispensable elements for maintaining homeostasis [37]. Cu ions may cause toxicity once they exceed the physiological tolerance range *in vivo* [38,39]. Therefore, the possible health effects and toxicology of CuO NPs have caused great concern to both the public and scientific researchers.

Toxicity assessment studies have primarily focused on investigating the effects of different exposure routes, such as the respiratory or gastrointestinal tracts. Yokohira *et al*. [40] performed lung carcinogenic bioassays following intratracheal instillation of CuO NPs. Histopathological assessment showed that CuO NPs induced severe acute inflammatory changes in the rat lung at high doses and chronically at low doses or with frequent instillations. Karlsson [41] reported that CuO NPs could cause cytotoxicity and DNA damage in the human lung epithelial cell line A549. Oxidative lesions were verified by measuring intracellular production of reactive oxygen species (ROS) with the oxidation-sensitive fluorescent probe, 2′,7′-dichlorofluorescin diacetate (DCFH-DA). Comparing the relationship between ROS generation and DNA damage, Xing and colleagues proved that oxidative stress was the primary toxic effect [42]. Compared to cells cultured in normal medium, cells exposed to CuO NPs exhibited reduced catalase and glutathione reductase (GR) enzymes activity and showed increased glutathione peroxidase (GPx) activity. The observed increase of the ratio of oxidation to total glutathione suggested that CuO NPs not only generated ROS, they also blocked cellular antioxidant defenses [43]. It was worth noting that the toxicity of CuO NPs *in vitro* was greater than that of many other metal oxide NPs and nanotubes. When branchial chloride cells were exposed to waterborne Cu, the percentages of apoptotic and necrotic chloride cells increased and intercellular spaces dilated and were invaded by large number of white blood cells [44].

Environmental research into CuO NPs’ toxicity has mostly focused on the effects on organisms, especially those in aqueous environments. The most common experiment models are algae and zebrafish, whose growth and toxicity are treated as environmental relevance indicators. Aruoja *et al*. [45] studied the toxicity of CuO NPs on the algae *Pseduokirchneriella subcapitata* using bulk formulation of metal oxide as a control. At low concentrations, CuO NPs (EC_50_ = 0.71 mg Cu/L) were more soluble and more toxic than the control (EC_50_ = 11.55 mg Cu/L). The results showed that the toxicities of bulk and nanosized CuO were largely influenced by soluble Cu ions. These findings were similar to the conclusions drawn by Grosell [46] and Griffitt [47]; those publications both proved that the soluble Cu forms were highly toxic to fish. Some studies also reported that CuO NPs’ suspensions might damage gill lamellae and inhibit epithelial cell proliferation by altering plasma metal levels [47], as well as chloride cell number and diameter [48]. Therefore, Gomes *et al*. [49] considered that mussel digestive gland could aggregate CuO NPs and result in toxicity.

The results of Shi [50] indicated that CuO NPs decrease chlorophyll content of the duckweed and that CuO NPs toxicity is three to four times higher than that of ionic Cu, because of the larger uptake of NPs-released Cu. Griffitt *et al.* [47] compared the responses of ﬁsh exposed to nanoCu solution and soluble Cu and reported that the effects of gill morphology and transcription were not solely due to the dissolution of Cu NPs. CuO NPs also had adverse effects on bacteria, and Cu^2+^ dissolving from CuO NPs induced toxic effects by triggering ROS production and DNA damage in bacteria [51].

### 2.2. Toxicity of ZnO NPs

The toxicities of CuO NPs, CuO bulk, and Cu^2+^ are different, but the 30-min half maximal effective concentration (EC50) of ZnO NPs indicate that the toxic effects of ZnO NPs, bulk ZnO particles and Zn^2+^ are similar [52,53,54].

The toxic effects of ZnO NPs on organisms were studied using different treatment routes. Because ZnO NPs are widely used in sunscreen, human skin exposure to ZnO NPs was one of the most important routes. Cross *et al.* [55] reported the dermal adsorption of ZnO NPs. When Franz-type diffusion cells were exposed to a novel, transparent nano-ZnO sunscreen formulation for 24 h, there was no sign of penetration of ZnO NPs penetration. Moreover, electron microscopy indicated that no NPs could be detected in the lower stratum corneum or viable epidermis. Oral, inhalation, and intratracheal instillation routes have also been used to evaluate the acute toxicity of ZnO NPs. Zheng *et al*. [56] assayed the toxicity of ZnO NPs in mice exposed via the digestive tract. Compared with the blank group, the spleen and brain cells were normal, whereas other primary organs (including heart, lung, liver, and kidney) were damaged. These results were supported by findings of Wang *et al.* [57], which showed that the pathological changes induced by ZnO NPs were both size- and dose-dependent. When mice were treated via the intratracheal tract, histopathological observation revealed serious pulmonary inflammation, proliferation, and alveolar wall thickening in the lungs of all the treated mice groups. Moreover, all of these changes were more serious in animals that received higher dosages.

ZnO NPs were strongly cytotoxic at lower concentrations and exhibited strong protein adsorption abilities [35], which may be contribute toward their cytotoxicity. Brunner *et al.* [58] found that almost all human or rodent cells died following exposure to ZnO NP concentrations above 15 ppm. Sharma [36] indicated that ZnO NP-induced cytotoxicity was concentration- and time-dependent. Other damage was induced by oxidative stress, which could be measured by increased hydroperoxide levels, depleted of glutathione level, and reduced catalase activity, all of which can contribute to cell death. Toxicity studies in *Caenorhabditis elegans* showed that ZnO NPs can inhibit growth and reproductive capability [59]. ZnO NPs can induce cytotoxicity by increasing oxidative stress in the human colon cancer cell line LoVo [60]; results indicated that ZnO NPs caused a time- and dose-dependent decrease of cell number compared with untreated cells, and reduced TMRM (Tetramethyl Rhodamine Methyl Ester Perchlorate) fluorescence was detected. ZnO NPs induced increased levels of hydrogen peroxide and hydroxyl radicals, decreased levels of molecular oxygen and glutathione, and reduced interleukin-8 (IL-8) release (a signal for proinflammatory mediator release). These findings indicate that ZnO NPs might be used for carcinoma therapy in the future.

The toxic effect of ZnO NPs on the environment has also been reported. Compared to bulk formulations, small NPs are more toxic to aquatic organisms. Lin *et al*. [61], measured plant root growth and seed germination following treatment with five types of metal and metal oxide NPs. The results showed that Zn and ZnO NPs obviously inhibited growth. Franklin *et al*. [54] reported that ZnO NPs inhibited alga *P. subcapitata* growth. Xiong *et al*. [62] proved that ZnO NPs acute toxicity to zebrafish was dose-dependent, and zebrafish death occurred once the concentration reached 2mg/L. Effects on endobenthic species were assayed through isotopically labeled ZnO NPs, and the results indicated that clam burrowing behaviors and feeding rates were significantly impaired after exposure to ^67^ZnO NPs [63]. To test and compare the acute toxicity of CuO and ZnO NPs to crustaceans species *Daphnia magna* and *Thamnocephalus platyurus*, and protozoan *Tetrahymena thermophila*, Blinova *et al*. [64] performed experiments in artificial freshwater and natural water. The results indicated that the half maximal lethal concentration (LC_50_) values of nano and bulk ZnO were lower than that of CuO NPs.

Another toxicity study showed that ZnO NPs exposed terrestrial isopods *Porcellio scaber* died following bioaccumulation [65]. Exposure to the different types of dietary Zn (unmodified ZnO NPs, Zn^2+^ ions and micro ZnO) showed that the amount of assimilated Zn in body and bioaccumulation factors were dose-dependent. This suggests that bioaccumulated Zn was primarily from Zn^2+^ dissolved from the ZnO NPs, not from the particles themselves. That might indicate metal oxide NPs’ dissolubility is an important component of the toxic effect.

Several reports indicated that ZnO NPs appeared to resist microorganisms [66], and the bacterial toxicity of them showed higher than that of the bulks [67]. In bacteria toxicity tests, ZnO NPs were thoroughly dispersed in a culture medium. According to the study of Hu *et al*. [68], ZnO NPs were most toxic to *E. coli* and had lower LD_50_ values than other metal oxide NPs [69]. This showed that the intrinsic toxic properties of metals played an important role in metal oxide NPs’ toxicity [70]. Some results [71] indicated that ZnO MPs strongly inhibited yeast growth, and Zn sensor bacterium showed that the toxic effect came from particle dissolution, which could disrupt cellular Zn homeostasis. Treated *E. coli* cells exhibited triple membrane disorganization and increased membrane permeability following exposure to ZnO NPs, which were internalized and accumulated in cell membranes [72]. Because they show antibacterial properties, low-cost ZnO NPs may be the next generation of efficient antibacterial agents.

## 3. Key Factors and Toxicity Mechanisms

To adequately understand CuO and ZnO NPs’ toxicity, it is essential to identify how theyinduce adverse biological effects. Although these investigations are ongoing, we discuss what is currently known below.

### 3.1. Key Factors

**Figure 2:**
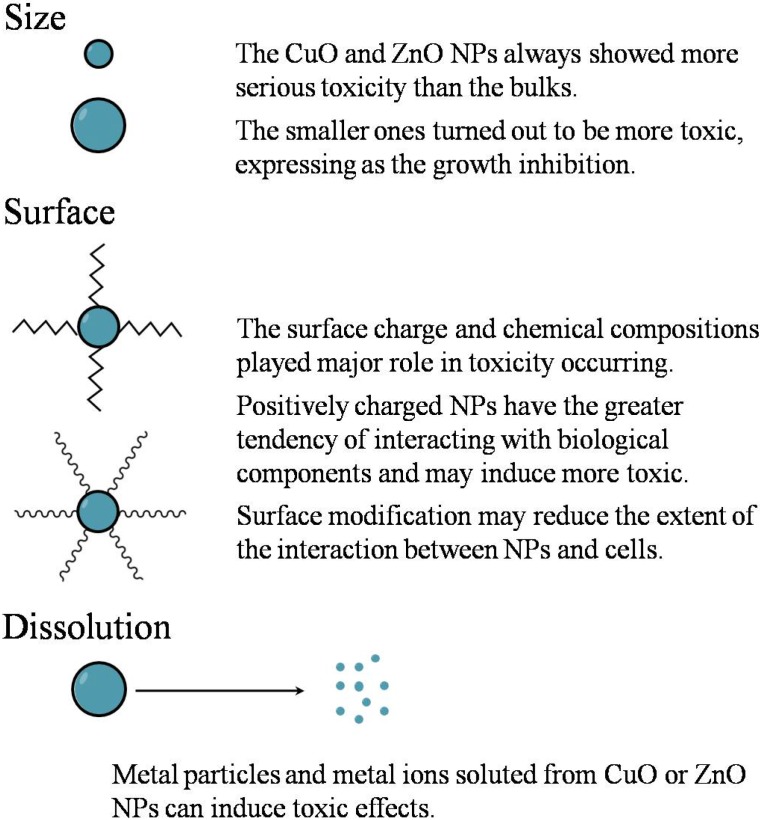
Schematic overview of relevant NPs’ characteristics that can be controlled to influence toxicity: size, surface modification, and solubility. Please see the main text for further details and interpretations.

The key factors in hazard evaluation after exposure to bulk materials are chemical composition, dose, and exposure route. However, for nanomaterials, additional factors include, nanosize, nanosurface, dissolution, self-assembly, quantum effects, nanostructures, concentration, and aggregation [73]. It is of great importance to understand how these factors affect nanomaterial toxicity; ideally, it should be so low that they do not damage organisms or their environments. For ZnO and CuO NPs, the main factors considered are size, surface characteristics, dissolution, and exposure routes (Figure 2).

#### 3.1.1. Size Factor and Surface Characteristics

It is well known that most of the novel properties of nanomaterials are related to their size. Several studies have reported that nanosized particles always showed more serious toxicity than bulks [71] and suggested that size was one of the key factors influencing the toxic effect of NPs. Hund-Rinke *et al*. [74] exposed green algae *Desmodesmus subspicatus* to two products with particle sizes of 25 and 100 nm and found that the smaller ones inhibited growth to a greater degree. Smaller nanosized particles also caused thrombocyte and granulocyte activation and hemolysis and induced inflammation and hemolysis in human blood samples [75].

The size of NPs is directly correlated to many essential properties, such as surface property, solubility, and chemical reactivity, and some of them have effects on the interactions between nanomaterials and biomolecules that subsequently affect the nanotoxicological behaviors of NPs *in vivo* [76]. For example, decreasing size results in increasing NPs’ specific surface area, which promotes not only the accumulation of NPs, but also an increase of reactivity and enhanced interactions between NPs and biomolecules. Because of their tiny size, NPs can cross the small intestine by persorption and further distribute into the blood, brain, kidney, and liver [77,78]. Varying NP radius size results in different cell uptake rates [79], which affect toxicity. Because they are not efﬁciently phagocytized by macrophages, NPs are poorly cleared *in vivo* [80]. With respect to toxic effect on the lungs, smaller particles can be deposited deeper, causing greater damage. It also was reported that toxicity was related to dimension, with one-dimensional structures showing greater toxicity [81].

Besides size, surface characteristics (e.g., surface charge and surface defect) of NPs are other key factors that determine CuO and ZnO NPs toxicity. Hwang and colleagues [68] indicated that metal oxide NPs’ cytotoxicity was related to the surface charge, and the toxic effect to bacterium reduced when cation charge increased. It can be explained by speculation about electrovalent attraction that zincative bacterium particles could be attracted by cation surface of metal oxide NPs, with lower charged particles easier to be associated. This is also the case when positive particles can be directly adsorbed onto negatively charged proteins such as albumin. Even if particles have a negative charge, they can absorb onto albumin via mediating cations such as calcium (Ca^2+^) [35]. Alternatively, Berardis *et al*. [60] compared the cytotoxicity between ZnO and TiO_2_ NPs of the same size and found that they showed differential effects to marine algae, which suggested that their chemical compositions also played a major role. The surface properties of NPs can be adjusted via the functionalization of NPs with functional molecules [1,82]. These functionalized NPs have better dispersity in aqueous solutions, which prevents the loss of most of the size-dependent effects. If surface properties cannot be controlled, NPs might quickly agglomerate into larger particles and easily interact with biomolecules and organs, possibly resulting in toxicity [83,84]. More studies should be done to improve the synthesis and functionalization of NPs to make better use of metal oxide NPs while reducing toxic effects to human and environment.

#### 3.1.2. Dissolution

Another key factor of toxicity is the dissolution of metal ions. Studies have suggested that the cell walls of plant roots and leaves excluded large NPs [85,86] but absorbed solute metal ions from NPs. Solubility is an important property that explains the reasons of toxic effects on many organisms. Brunner *et al*. [58] found that the six-day toxic effect of soluble metal/metal oxide NPs was higher than insoluble ones applied at the same concentration. Algae growth is sensitive to dissolved Zn [87], and Cu-sensor bacteria demonstrated that CuO NPs were much more soluble than bulk CuO [53]. Because nanosized particles have larger surface areas to interact with solvent molecules than bulk ones with the same weight and NPs show faster dissolution. Zn and ZnO NPs solubility is higher than that of bulks [87,88]. Thus, nanosized particles are more toxic. This is also a possible reason to explain the toxicity of CuO NPs, which exceeded that of bulk CuO by about 40-fold. Similarly, CuO NPs were 45- or 50-fold more toxic than bulk CuO [64]. In natural water, metal oxide NPs can exist as aggregates, which is likely driven by the divalent ions and the low zeta potential [47]. When agglomerates form a neck between two or more particles, they create an area of negative surface curvature, and nucleation is predicted to occur at this interface under equilibrium conditions. This can result in fusion of the agglomerate and a reduction in total particle surface area.

The solubility of ZnO and CuO NPs is related to solution pH and temperature. Palmer and Bénézeth reported that the solubility curve of CuO in water as function of pH was a V-shaped profile at a specific temperature, and pH values from 9 to 11 have little effect on solubility at ambient temperature [89]. As a result, CuO NP toxicity is related to solubility, which is similar to that observed for CuO bulks. The toxicity curve of CuO NPs in water as a function of pH can be deduced based on the solubility curve of CuO in water. CuO NPs show the least toxicity when pH is between 9 and 11, and the toxicity is nearly constant. A one-unit shift in pH on either side of the minima can induce an extreme increase of toxicity. Temperature changes have similar effects on the toxicity of CuO NPs.

However, the role of solubility on nanotoxicity is disputable. It was reported that release of metal ions from NPs was minimal, and such low concentrations of Cu or Zn ions did not cause any cytotoxic effects [90]. Through comparing the toxicities of three different metal oxide NPs (Fe_2_O_3_, Y_2_O_3_, and ZnO) with different concentration exposures, Gojova *et al*. [91] proved that the toxic effect in vascular endothelial cells was due to chemical compositions rather than solubility. Midander *et al*. [92] demonstrated that Cu can be released from CuO NPs, and the soluble quantity was different in various media. Cytotoxicity studies showed that the CuO NPs exerted toxic effects, and the released fraction of Cu from CuO NPs only accounted for a little part of this toxicity. Moos’s group [90] observed that the robust hallmarks of apoptosis emerged in RKO cells treated with ZnO NPs but not soluble Zn salt. The toxicity in cells was deemed to depend on contact with NPs rather than exposure to soluble metal ions solutions. However, the researchers were careful to mention that this may be dependent on the cell types used. When extending exposure time to 24 h, Cu^2+^ released from CuO NPs caused significant DNA damage and accounted for 31% of membrane damage [42]. It is believed that both metal oxide NPs and dissolved ions will contribute to cytotoxicity, when cells are exposed to metal oxide NPs; the potential toxic effects of CuO and ZnO NPs are the combined effects of particles and soluble.

Blinova [64] has also shown that the toxicity of Cu ions in river water containing higher dissolved organic carbon (DOC) was lower than that in river water with lower DOC content. It indicated that natural waters might have higher mitigation effects for toxicity of CuO NPs than bulk CuO or ZnO, because of the strong complexing of Cu to natural organic matter to reduce bioavailable Cu ion concentrations. This also enlightens us regarding a possible solution to decrease the toxicity of metal oxide NPs.

#### 3.1.3. The Importance of Exposure Routes

Different exposure routes can result in various effects. Metal oxide NPs can be administered via intratracheal (pulmonary toxicity), oral, nasal, skin or other routes [73]. Whether they can diffuse in the respiratory tract is primarily determined by the thermal motion of air molecules from inhaled and exhaled air. Diffusion motion affects NP character and physiology and influences particle deposition. Particles can translocate into the blood and other organs from the respiratory tract and further induce certain lesions [77,93]. ZnO and CuO NPs can also induce eosinophilic to severely cytotoxic inflammation, and eosinophils play a key role in mediating asthma and other allergic conditions [94]. Meanwhile, some studies have shown that NPs can access the brain through intranasal instillation [95,96], which avoids the suppressive effect of the blood–brain barrier (BBB). Via this route, tiny particles or metal ions enter systemic circulation directly from the nasal mucosa, enter the olfactory bulb via axonal transport along neurons, and directly access the brain via the olfactory epithelium [95]. Metal oxide NPs could be transferred into brain vessels and tissues in this way, which could increase bioavailability. At the same time, this might also lead to toxic effects and inflammatory responses in the brain [77] and central nervous system damage. In the gastrointestinal tract, exogenous substances diffuse and pass into the villi, intestinal enterocytes, and epithelium to enter systemic circulation. Moreover, gastric juice supplies a strong acid ambience (pH~2). In this environment, metallic and metal oxide NPs are dissolved into metal ions, which can freely penetrate cells via ion channels and biological pumps. Once the bearable range is exceeded, toxic effects may occur. Toxicities caused by metal oxide NPs were diverse in different environments. It is well known that dissolved Cu NPs can inhibit Na^+^/K^+^-ATPase activity, resulting in ionoregulatory toxicity [44]. Chen *et al*. [97] reported that the toxicities of Cu NPs to gastric tissues and kidney were caused by increasing H^+^ and massive production of HCO_3_^−^. For metal oxide NPs, blood capillaries and lymphatic tissue play important roles in absorbability, and NPs entering blood capillaries or lymphatic tissue may be carried to other organs where they accumulate. A test of inflammation induction in vascular endothelial cells indicated that NPs entering vessels can lead to considerable cell toxicity, in addition to a pronounced inﬂammatory response.

### 3.2. Toxicity Mechanisms of ZnO and CuO NPs

**Figure 3:**
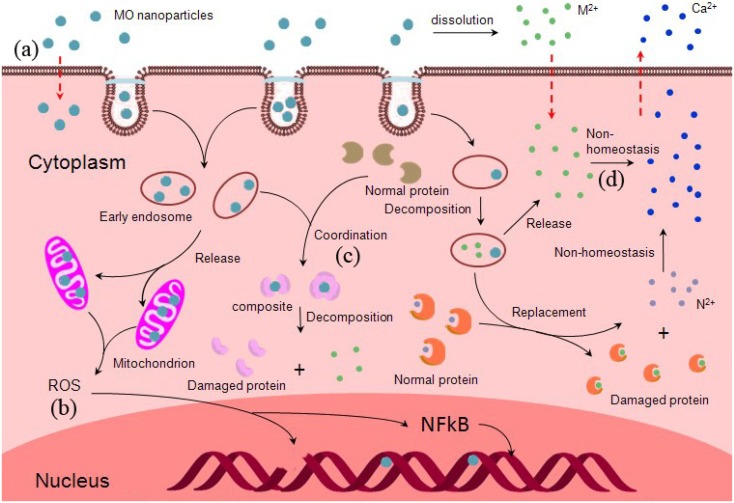
Schematic overview of the different pathways inducing cellular toxicity by CuO and ZnO NPs. **(a)** Potential mechanisms of CuO and ZnO NPs’ entry into cells; **(b)** The ROS effect of intracellular CuO and ZnO NPs; **(c)** The coordination effect of Cu^2+^ and Zn^2+^ released from NPs in cell; **(d)** The non-homeostasis effect disrupted by Cu^2+^ and Zn^2+^.

It is known that both ZnO and CuO NPs are able to cause toxicity in organisms and the environment. The key factors of determining toxicity were briefly described above. Though this field is in an early stage, some important toxic data have been gathered. Nevertheless, the precise mechanisms of toxicity remain unknown. To achieve safe utilization of NPs, more studies must be performed. The toxicity mechanisms of CuO and ZnO NPs may be mainly dependent on the interaction between NPs and biomolecules, and toxicity mainly involves protein unfolding [98], ﬁbrillation, thiol cross-linking, and enzymatic activity loss. Below we discuss three mechanisms that potentially explain why CuO and ZnO NPs exert toxic effects: oxidative stress, coordination effects, and non-homeostasis effects (Figure 3).

The mechanism of CuO and ZnO NPs’ entry into cells is shown in Figure 3a. NPs candiffuse across the membrane occurs directly when the size is small enough, there are positive ions on the surface of NPs, or when other variables are present [99,100]. Meanwhile, ion channels and transporter proteins permit NPs to cross the plasma membrane. Some NPs enter cells via “endocytosis”: the membrane wraps around them, and vesicles transport NPs into cells. Cu^2+^ and Zn^2+^ solute from NPs can enter cells by transport and ion/voltage-gated channels [101,102]. Intracellular ROS effect induced by CuO and ZnO NPs is shown in Figure 3b. NPs can directly interact with oxidative organelles such as mitochondria, redox active proteins stimulate ROS production in cells, and ions (Cu^2+^, Zn^2+^) produced by NPs can induce ROS by various chemical reactions. ROS can induce DNA strand breaks, and affect gene expression. Moreover, Cu^2+^ and Zn^2+^ ions have the ability to form chelates with biomolecules or dislodge the metal ions in specific metalloproteins, which may results in functional protein inactivation (Figure 3c). Cu^2+^ and Zn^2+^ released by NPs increase their local concentration and disrupt cellular metal cation homeostasis to result in cell toxicity (Figure 3d).

#### 3.2.1. Oxidative Stress

ROS generation and oxidative stress induction are the major toxicological mechanisms of ambient NPs. Large amounts of ROS could be generated even when only small amounts of CuO or ZnO NPs are incorporated into cells [103]. How ROS are induced by the metal oxide NPs will be discussed in this section. NPs can induce ROS directly, once they are exposed to the acidic environment of lysosomes [104] or interact with oxidative organelles, such as mitochondria [105]. NPs are able to interact with biomolecules due to their large specific surface area that endows CuO or ZnO NPs with high reactive activity and electronic density [106]. During this process, chemical reactions take place and increase superoxide radical (O^2−^) formation, which leads to ROS accumulation and oxidative stress [60]. ROS are oxygen derivatives that arise during life in an oxygenated environment and contain superoxide anions (O^2−^), hydroxyl radicals, and hydrogen peroxide. ROS can react with biomolecules, causing an imbalance between the production of reactive oxygen and the biological system’s ability to detoxify reactive intermediates or repair the resulting damage [107].

NP-induced ROS production can lead to a range of biological responses that depend on the relative abundance of ROS production, the type of cellular pathways, and the antioxidant response element that are engaged in oxidative stress [108]. The oxidative stress induced by metal oxide NPs was studied by exposing zebrafish and cells to NPs. The results showed that the quantities of ·OH in metal oxide NPs’ suspensions were much higher than in bulk formulations, and the oxidative stress and oxidative damage generated in the absence of light [62].·OH is generally considered as one of the most toxic ROS species and is able to oxidize almost all the cellular components [109]. The extracellular OH generated by metal oxide NPs might induce oxidative damage on cell membranes, which can cause toxic effects in organisms.

It has been proven that oxidative stress represents a common mechanism for cell damage induced by NPs [110], and the mechanism has been validated in many NPs’ toxicity studies [107]. Upon entering the cell, particles may induce intracellular oxidative stress by disturbing the balance between oxidant and anti-oxidant processes. Excessive oxidative stress may also modify proteins, lipids, and nucleic acids, which further stimulates the anti-oxidant defense system or even leads to cell death. Meanwhile, with increased ROS production, NPs can cause DNA damage and increase gene expression of the death receptor [107]. In addition, increased ROS induced by NPs in lysosomes can cause DNA point mutations or induce single- or double-strand breaks [111]. Another major oxidative stress response is intracellular Ca^2+^ release, which leads to mitochondrial perturbation and cell death [108]. Many diseases, such as lung, cardiovascular, and autoimmune diseases, as well as aging [109], have been linked to oxidative stress.

#### 3.2.2. Coordination Effects

Interactions between metal oxide NPs and proteins *in vivo* or *in vitro* involve coordination and non-covalent interactions [112]. Protein binding to ZnO NPs can result in major structural changes, unfolding behavior, of the periplasmic domain of the ToxR protein by a substantial decrease in the α-helical content of the free protein [98]. Metal cations assist in protein folding or bind to folded proteins to enhance, diversify, or reduce their function. Approximately 40% of all known proteins contain metal cations, and Cu^2+^ and Zn^2+^ ions bind to proteins under physiological conditions [113,114]. If mutations occur in existing metal-binding sites, protein structure and activity may be reduced or lost [115]. It has been discussed that solubility is one of the key factors that causes toxicity. Cu^2+^ and Zn^2+^ ions have the ability to form chelates with donor molecules. A large number of biomolecules in organisms contain coordination atoms, mainly O and N atoms, which can donate lone electrons to form chelates with Cu^2+^ and Zn^2+^. The coordination atoms of biomolecules are almost always located in active sites. Therefore, coordinating with biomolecules may offer opportunities to inactivate biomolecules, largely destroying the functions that maintain normal physiology processes, and then subsequently result in toxicity. The affinity of a metal cation for a given ligand is governed by the metal’s charge, ionic radius/polarizability, and charge-accepting ability. Different metal ions have various binding sites and affinity with a functional protein [116]. Dudev and Lim [113] declared that when an Mg^2+^ ion binds with a specific protein, it could be dislodged by Zn^2+^. The reason may be that Zn^2+^ can accept more charge from the ligand than Mg^2+^ and that the Zn^2+^ complex is more stable. Coordination effects can directly and indirectly promote cellular DNA damage. NPs can induce physical damage to genetic material via direct interaction with DNA or DNA-related proteins because NPs can diffuse through the nuclear pore complexes or gain access when the nuclear membrane dissolves during mitosis if the NPs are small enough [111]. Proximal perinuclear NPs can hinder cellular transcription and translation machinery. Released metal ions may also lead to cytoplasmic mRNA degradation by interacting with mRNA stabilizing proteins [117], which contain metal responsive domains. NPs can interact with cellular signal molecules, which lead to signaling cascade activation [118] and induce DNA damage and cell death.

#### 3.2.3. Non-Homeostasis Effects

There are many important roles for functional metals *in vivo*. Some metals function independently and the others keep the composite functioning. Cu^2+^ and Zn^2+^ ions play significant roles in maintaining organisms homeostasis [37], and low or high levels of Zn and Cu can disrupt homeostatic mechanisms. If homeostasis variation exceeds the range of physiological tolerance, toxicity occurs. In addition, CuO and ZnO NPs may release Cu^2+^ and Zn^2+^, respectively, which increases the local concentrations of metal ions and can disrupt metal cation cellular homeostasis. It has been reported that the oxidative stress can stimulate the increase of the intracellular Ca^2+^ concentration [108], altering intracellular Ca^2+^ flux, and Ca^2+^ homeostasis has a role in the proinflammatory effects of ultra-fine particles. In addition, intracellular Ca^2+^ has been implicated in the control of a large variety of cellular processes [119]. Zn sensor bacteria showed that toxic effects occurred following particle dissolution, which could disrupt cellular Zn homeostasis [71].

## 4. Conclusions

We have discussed the toxicity of CuO and ZnO NPs, and the corresponding mechanisms. NPs exhibit greater toxicity than micro ones with the same composition, and various-sized NPs induced different levels of cytotoxicity and DNA damage [120]. Metal ions that dissolved from metal oxide NPs also play an important role in inducing toxicity [42], and the character of the exposed environment influences toxic effect because it affects the state of NPs. Oxidative stress, coordination effects, and non-homeostasis effects were discussed in the context of understanding the toxicity mechanisms of metal oxide NPs. Relating protein affinity to NPs would pave the way for NPs’ use as bio-sensors and in drug delivery [121]. ZnO NPs induce cause tumor cell cytotoxicity and synergistically enhance the chemotherapeutic agents action [122], suggesting potential application in curing cancers. This and other attractive properties open up an avenue to increase the applications of CuO and ZnO NPs in many fields, including biomedicine and catalysis. To ensure that NPs are safe to organisms and the environment, toxicity must be decreased to the non-significant level. This objective requires further work that focuses on toxicity factors of metal oxide NPs. Reducing these factors will weaken toxicity mechanisms. Groundbreaking work on controlling NP diameter [79] and surface modification should decrease toxic effects [121]. Alternatively, redirecting toxic effects to target tissue, regulating the release of metal ions from metal oxide NPs [123], and proper exposure to route selection, will also reduce the toxicity of metal oxide NPs.
